# General practice chlamydia testing: a qualitative study of staff approaches using behavioural change theory

**DOI:** 10.3399/BJGP.2024.0498

**Published:** 2025-07-29

**Authors:** Amna Asad, Beattie RH Sturrock, Jessica Carter, John M Saunders, Jackie A Cassell, Greta Rait, Lorraine K McDonagh

**Affiliations:** 1 Research Department of Primary Care and Population Health, University College London (UCL), London, UK; 2 Institute for Global Health, UCL, London, UK; 3 Migrant Health Research Group, Institute for Infection and Immunity, City St Georges, University of London and Wolfson Institute of Population Health, Queen Mary University of London, London, UK; 4 UK Health Security Agenda (UKHSA), Institute for Global Health, UCL, London, UK; 5 Department of Primary Care and Public Health, Brighton and Sussex Medical School, Brighton, UK; 6 Research Department of Primary Care and Population Health, UCL London, London, UK; 7 National Institute for Health and Care Research Health Protection Research Unit in Blood Borneand Sexually Transmitted Infections at UCL in Partnership with UKHSA, London, UK

**Keywords:** chlamydia, general practice, behaviour change

## Abstract

**Background:**

Chlamydia is the most diagnosed bacterial sexually transmitted infection (STI) in England, but opportunistic testing remains low in general practice despite high prevalence among young people. Attempts to increase testing have been met with little success; therefore, there is a need to explore why rates remain low and how this may be improved.

**Aim:**

To explore general practice staff perceptions of opportunistic chlamydia testing, including barriers, facilitators, interventions, and policies, using the Behaviour Change Wheel (BCW).

**Design & setting:**

Qualitative interviews and focus groups were undertaken with general practice staff in England.

**Method:**

Twenty-three semi-structured individual interviews and seven focus groups with general practice staff were conducted. Data were analysed using inductive thematic analysis, followed by thematic categorisation onto the BCW.

**Results:**

Participants identified several barriers to chlamydia testing corresponding with BCW components, including low perceived knowledge (psychological capability), general practice context (physical opportunity), cultural norms (social opportunity), testing not prioritised (reflective motivation), and concerns about patient reactions (automatic motivation). Proposed intervention functions included education, persuasion (for example, posters), incentivisation (for example, financial incentives), and environmental restructuring (for example, computer reminders). Potential policy categories discussed were communication and marketing (for example, campaigns) and service provision (for example, GP drop-in sessions at other venues).

**Conclusion:**

This study identified barriers to chlamydia testing in English general practice and potential ways to address these issues, contributing new insights to existing literature. This research can be utilised to design multi-component, impactful interventions to increase testing in general practice and ultimately reduce harm posed by chlamydia infections.

## How this fits in

Chlamydia testing in English general practice remains low despite National Chlamydia Screening Programme (NCSP) guidance (that is, opportunities to screen young women are missed and screening of young men persists despite policy changes). Previous research on staff perspectives has identified barriers to testing in general practice yet attempts to overcome these barriers and increase testing have largely been unsuccessful. This research provides perspectives of chlamydia testing in general practice by primary healthcare staff mapped onto behaviour change theory. These findings may guide the creation of theoretically informed interventions to increase testing in general practice and help tackle a key population health problem.

## Introduction

Chlamydia is a global health concern with substantial economic and social costs. Worldwide, an estimated 129 million new infections occur each year.^1^ In England, chlamydia is the most diagnosed sexually transmitted infection (STI) with 194 970 diagnoses in 2023.^2^ It is most prevalent in young adults,^2^ is often asymptomatic, and if untreated, can lead to transmission and morbidity such as pelvic inflammatory disease, ectopic pregnancy, and tubal factor infertility.^3,4^

The US Centers for Disease Control and Prevention recommends annual chlamydia screening for sexually active women, transmen, and gender diverse people with a cervix aged <25 years, women aged >25 years at increased risk, pregnant people during first prenatal visit, and sexually active men in high-prevalence settings.^5^ In Europe, most countries monitor chlamydia, some have screening programmes (Germany, Sweden) but these are not national.^6,7^ Only England has a National Chlamydia Screening Programme (NCSP; rolled out 2003–2008), which initially offered opportunistic chlamydia testing to all sexually active <25-year-olds. In 2021, the focus shifted to screening women and people with a womb and ovaries, aiming to reduce untreated complications (rather than prevalence).^8^ This is because including men was not ‘*cost-effective in preventing chlamydia-related harms*’^9^ and owing to antimicrobial resistance concerns.^10^ Men and women can still access testing in specialised sexual health services, and the NCSP offers testing in other settings, including general practice, pharmacies, and online.^11^

In England, general practice is a primary care service that comprises multidisciplinary teams (including, for example, GPs, nurses, pharmacists, mental health practitioners), and often serves as the first point of contact for patients, treating common medical conditions, and referring patients to secondary services if needed.^12^ Young people have indicated acceptability of tests in GP surgeries.^13–17^ However, despite NCSP efforts, general practice testing remains low. Among women in 2023, 17.4% of tests^17^ were from general practice, while 46.9% were via the internet (the most common setting).^2^ Commonly reported staff barriers include time constraints, low perceived knowledge of testing and treatment, and discomfort discussing sexual health.^13,14,18–23^ Barriers for young people include concerns over self-sampling, lack of awareness, perceived low risk, embarrassment, lack of NHS resources, getting appointments, and time constraints.^13^

### Behaviour Change Wheel

**Figure 1.**
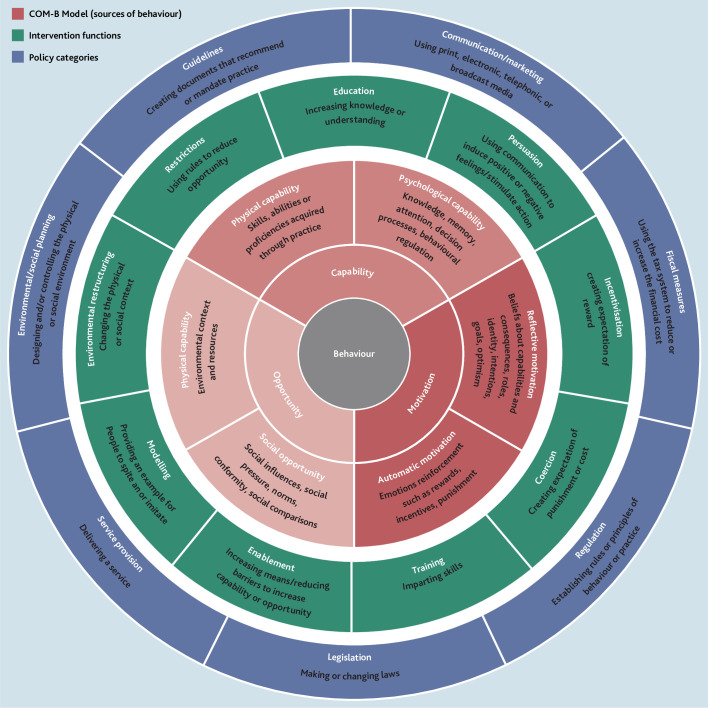
The Behaviour Change Wheel. Reproduced under a Creative Commons (CC BY) license from Michie *et al.* ^32^

Financial incentives and educational interventions in the UK and Australia, have been unsuccessful or changed behaviour only temporarily,^24,25^ potentially owing to lack of theory used.^26–32^ Change in practice requires theory-guided behaviour change interventions.^31,32^ The Behaviour Change Wheel (BCW) is a meta-theoretical framework used to design behaviour change interventions (Figure 1).^32^ The three-tiered tool identifies internal mechanisms that promote behaviour change, outlines levers to support this, and can considerably change behaviour.^33–35^ For example, a primary care-based randomised control trial of a BCW informed-educational intervention on alcohol screening and brief interventions found significantly increased screening activity compared with waiting list control.^36^

The centre of the BCW is the COM-B model, a theory that proposes behaviour to be an interaction between capability, opportunity, and motivation.^14^ The second tier includes interventions through which behaviour can be changed. The third outlines policy to support interventions, such as guidelines, legislation, and fiscal measures.

### Current study

The aim of this study is to identify the following: 1) barriers to chlamydia testing for general practice staff; 2) interventions to overcome identified barriers; and 3) policy categories to support these interventions.

## Method

This study is reported in line with the consolidated criteria for reporting qualitative research (COREQ) checklist.^37^

### Participants

Participants were healthcare professionals working in general practice: general practitioners (GPs; *n* = 31), practice nurses (*n* = 25), GP registrars (*n* = 7), healthcare assistants (*n* = 5), nurse practitioners (*n* = 4), administrators (*n* = 2), and a senior dispenser (*n* = 1). See Box 1 for role definitions.^38–44^

Box 1. **Role definitions**

**Administrator**

Ensures smooth operation of the practice by handling various non-clinical, organisational tasks (for example, reception duties, appointment scheduling, record management). Administrators in this study did not have direct patient contact^^39^^

**GP**

Primary care doctors who treat common medical conditions and refer patients on for secondary care treatment^40^

**GP registrar**

Qualified doctors currently undergoing supervised specialty training to become a fully qualified GP, also known as GP trainee, GP registrar^^41^^

**Healthcare assistant**

Perform essential, routine tasks that support patient care (that is, taking observations, assisting with clinical procedures) under the guidance of nurses^^42^^

**Nurse practitioner**

Have extended scope compared with practice nurses and can work autonomously, have completed further training and have specialist experience^^43^^

**Practice nurse**

Provide a range of routine and preventive care, usually in coordination with GPs or nurse practitioners for more complex cases^^44^^

**Senior dispenser**

Responsible for managing the dispensing of medications and overseeing dispensary operations. Not trained as pharmacists yet require specialised training, work under the supervision of a registered pharmacist, also known as senior pharmacy assistant, senior dispensers^^45^^

Purposive and convenience sampling methods were used, including contact through practices, the National Institute for Health and Care Research (NIHR) Clinical Research Network, mailing lists, social media, and snowballing. For purposive sampling, recruitment was monitored to ensure a range of roles, demographics, and sexual health experiences were captured.

### Procedure

Semi-structured interviews (*n* = 23; 14 telephone, nine in-person) and focus groups (*n* = 7 in-person, see Supplementary Table S1 for group composition) were conducted.

Topic guides (Supplementary Box S1) were iteratively developed by the multidisciplinary team and piloted at University College London (UCL) with GPs. Two cisgender women (the corresponding author, a healthcare services researcher and the third author, an academic GP) conducted data collection. Only the research team and participants were present. Participants self-selected locations either in clinics or in private UCL rooms. Some participants were colleagues of the third author; where possible the corresponding author interviewed these people. Where this was not possible, the third author ensured participant comfort. Field notes were used to provide context. Participants received consent forms and an information sheet before participation, none dropped-out.

Interviews and focus groups lasted ~1 hour (interviews: 33 minutes, range = 21–56 minutes; focus groups: 41 minutes, range = 39–46 minutes). Conversations were audiorecorded, transcribed verbatim, checked for accuracy, and de-identified. Participants did not check the transcripts and only participated once. Participants were offered a £20 voucher as a thank you. Data collection continued while the emergence of new information was monitored and discussed at team meetings to ensure data saturation was reached.^45,46^

### Data analysis

A reflexive thematic analysis was conducted by the first author using Braun and Clarke’s following guidelines:^47^ 1) reading and re-reading transcripts to increase familiarity; 2) generating initial codes aligned with research objectives using NVivo (version 12; inductive coding); 3) grouping codes into themes and sub-themes; 4) reviewing and refining the thematic list; and 5) thematic categorisation onto the BCW after analysis (deductive coding). Themes were reviewed independently by two of the authors and findings were continuously discussed in team meetings where minor discrepancies (for example, codes did not fit as ‘neatly’ on the BCW) were resolved. To illustrate, ‘reactive approach’ was initially classified under social opportunity given primary care culture; after discussion, it was categorised under physical opportunity.

### Reflexivity

We considered our role in shaping the research, for example, our cultural (first author is from a country that has a healthcare system that prioritises preventive care), positional (third author is a female academic GP interviewing GPs), and professional (the corresponding author has a background in social and health psychology) assumptions. Being cognisant of our potential perspectives enabled improved probing and avoidance of premature interpretation (particular risks for clinical interviewers).^48^

## Results

Participants were predominantly GPs, female, White British, and working in urban locations (Table 1). Thematic maps and illustrative quotes are in Supplementary Box S2 (barriers), Supplementary Box S3 (intervention functions), and Supplementary Box S4 (policy categories).

**Table 1.** Sociodemographic characteristics and role definitions for study sample

Demographics	Interviews(*n*=23)	Focus groups(*n*=52)
**Role^a^**		
Administrator	0	2
GP	11	20
GP registrar	3	4
Healthcare assistant	0	5
Nurse practitioner	3	1
Practice nurse	6	19
Senior dispenser	0	1
**Age, years**		
18–29	2	5
30–49	14	27
50–64	7	11
≥65	0	5
Did not respond	0	4
**Gender identity**		
Male (including transmen)	5	12
Female (including transwomen)	18	38
Non-binary or gender diverse	0	0
Prefer to self-describe (open text)	0	0
Did not respond	0	2
**Ethnicity**		
Asian or Asian British		
Indian	3	7
Pakistani	1	1
Bangladeshi	3	1
Any other Asian background	2	2
Black or Black British		
Caribbean	0	0
African	0	3
Any other Black background	0	0
Chinese or other ethnic group		
Chinese	0	2
Any other ethnic group	0	2
Mixed		
Mixed: White and Black Caribbean	0	0
Mixed: White and Black African	0	0
Mixed: White and Asian	1	1
Any other Mixed Background	1	0
White		
White–British	10	25
White–Irish	0	1
Any other White background	2	6
Did not respond	0	1
**Years since qualification**		
<5	6	13
5–10	4	6
10–15	4	9
15–20	2	7
>20	7	12
N/A	0	5
**Years in general practice**		
<5	6	15
5–10	5	11
10–15	4	7
15–20	2	8
>20	3	11
N/A	3	0
**Previous experience in sexual health**		
Yes	11	18
No	12	34
**Size of practice by patient list**		
<5000	3	2
5000–10 000	7	31
10 000–15 000	8	17
15 000–20 000	1	0
>20 000	4	2
**Size of practice by number of GPs**		
Small <3	1	1
Medium 3–5	7	19
Large >5	15	32
**Location**		
Rural	10	14
Urban	13	38

a See Box 1 for role definitions.

### Barriers: sources of behaviour

Themes and sub-themes of barriers to testing were applied to the COM-B model (psychological capability, physical opportunity, social opportunity, reflective motivation, and automatic motivation).

### Psychological capability

#### Low perceived knowledge

Participants attributed lack of testing to low perceived knowledge of various aspects of chlamydia care, including prevalence, when to test, interpretation of results (and addressing positive results), and next steps (for example, partner notification):

‘*It’s a lack of familiarity with the equipment, with what you’re meant to order, when you’re meant to do the tests, what the test result means, what the local antibiotic policies are, how to do partner notification. It’s probably every step of the process as well ...’* (GP, female aged 31–40 years, interview 23)

Some providers were unsure whether testing was the responsibility of general practice or sexual health services, suggesting unfamiliarity with NCSP guidance: *‘It’s been confusing working out where patients should be best seen’* (GP, female, aged 31– 40, interview 6). Others highlighted forgetting to opportunistically test during busy and non-sexual health-related consultations.

### Physical opportunity

#### General practice context

Some barriers related to general practice context, including time constraints. Many providers emphasised they lack time to test within 10-minute appointments, being *‘constantly under pressure for time’* (GP, female, aged 41– 50 years, interview 13). Staff tended to prioritise patients’ presenting problems and not incorporate prevention into consultations, perhaps reflecting the wider healthcare context of a reactive approach to care.

Some preferred referring patients to sexual health clinics because they had a more comprehensive process, with full testing capabilities and partner notification. Some participants stated that lack of general practice resources for testing could be owing to funding cuts. One responder spoke of commissioners’ (that is, person responsible for planning and purchasing healthcare services for local populations) disinterest in funding chlamydia work, instead prioritising other prevalent, high-cost long-term conditions:

*‘We did some promotion a couple of years ago about chlamydia screening, but the funding came to an end so that stopped ... We’re really focusing on the high-cost areas: so diabetes and lung disease ...’* (GP, male, aged 51– 60 years, interview 7)

Multiple participants noted the lack of sexual health services for underserved populations. This included people living in rural areas and LGBTQIA+ (lesbian, gay, bisexual, trans, queer/questioning, intersex, asexual, plus other identities) patients: ‘[What] *is really sadly lacking are clinics, certainly rurally, for men who sleep with men’* (PN, female, aged 51– 60 years, interview 21). Participants cited the importance of having dedicated safe, non-judgmental spaces where patients can feel comfortable discussing sexual health and receive care tailored for their needs.

#### Testing process

In many cases, the testing process discouraged staff from testing, including patient discussions (for example, importance of testing, addressing stigma, obtaining informed consent, guidance on sample collection), data entry (for example, completing electronic or paper screening forms). These were perceived as time-consuming, emphasising staff’s time constraints:

*‘It takes a long time to put those packs together, physically actually putting the labels together, writing out the form, blah, blah, blah, blah, if I could just grab and it was all ready ...’* (GP, female, aged 31–40 years, focus group 4)

#### NCSP losing momentum

A few participants discussed NCSP efforts losing momentum over time, impacting practices because the NCSP no longer sent self-kits to assist with integration:

*‘I know, for a while, there was a big programme on opportunistically screening all young people, and we were sent kits in the post that we were to give out to young people to do their own chlamydia testing. But, that seems to have stopped, and then I haven’t heard anything about that for a long time now.’* (PN, female, aged 51–60 years, interview 16)

Staff expressed confusion over campaigns suddenly halting, highlighting perceived lack of communication from the NCSP.

### Social opportunity

#### Cultural norms

Cultural norm barriers included sex being seen as ‘*private*’ and societal hesitance to discuss it, with participants acknowledging its impact on the doctor–patient relationship and their comfort with discussing sexual health:

*‘I think sex isn’t something that we talk about regularly and as a culture … that does translate into like a doctor–patient relationship when perhaps* [it] *shouldn’t.’* (GP, female, aged 31–50 years, focus group 3)

Additionally, some staff stated offering testing to women is easier than men because they *‘could* [incorporate] *that conversation along with contraception’* (PN, female, aged 51– 60 years, interview 16). Staff also highlighted barriers to discussing tests with patients of the opposite gender. For example, a female GP felt uncomfortable offering tests to male patients while a male GP assumed that female patients would prefer female providers:

*‘Obviously, with sexual health, if it’s mainly females, often they like to see females. This can be restrictive for someone like myself.’* (GP, male, aged 21–30 years, interview 11)

### Reflective motivation

#### Belief that general practice should not test

A few participants believed that only sexual health clinics should test, demonstrating lack of support for NCSP’s guidance: *‘It should be done in the dedicated sexual health clinics, as opposed to in primary care ...’* (GP, male, aged21–30 years, interview 11).). As a result, providers choose not to test, referring patients to sexual health clinics for related services.

#### Perception that patients do not come to general practice

Most staff initially acknowledged serving a young demographic, but many later suggested they do not test because young people do not attend their practice. They cited various reasons, including patients’ reluctance to seek care and feelings of invincibility:

*‘They’re just a young group that think things will never happen to them. And they just don’t see their health as important, they’ve got a bit of a feeling that they’re invincible ...’* (NP, female, aged 61–70 years, interview 14)

Many providers expanded that they believe male patients visit less frequently than females, therefore, offering testing to them less. They attributed this to men delaying care, only coming in when *‘they feel truly unwell’* (Advanced nurse practitioner [ANP], female, aged 41– 50 years, interview 20).

#### Not testing in unrelated appointments

Some participants do not test in ‘*unrelated*’ appointments, with one GP describing it as *‘impossible’* to broach testing for an unconnected complaint such as a headache. Another staff member found it inappropriate:

*‘If ... you’re very distant from steering it over towards sexual health ... I’d probably feel a bit like it wasn’t appropriate actually to turn around and say, “Oh by the way, can you just do this chlamydia test for me?”’* (GP, male, aged 41–50 years, focus group 3)

Instead, providers prefer to introduce testing in similar contexts such as *‘consultations about pill checks, periods’,* and cervical screening.

#### Testing not prioritised

Some providers expressed low prioritisation of chlamydia testing because they have other items to cover in their consultations: *‘Chlamydia sort of tends to drop off the bottom when there’s so many other priorities’* (GP, female, aged 61–70 years, focus group 3). However, staff stated that while they do not opportunistically test, they would test at the patient’s request.

### Automatic motivation

#### Concern of patient reaction

Multiple responders cited fear of offending and embarrassing patients. Additionally, responders did not want to catch patients off-guard in unrelated appointments:

*‘... If it was totally unrelated to their presenting complaint then I guess that might make me a bit more reluctant ... You’re sort of catching the person a bit off-guard and they’re therefore not expecting you to ask them to talk about sexual health screening ...’* (GP, female, aged 21–30 years, interview 3)

However, one participant stated that patients are *‘aware’* and expect offers owing to testing outreach in schools.

### Intervention functions

Participants’ proposed facilitators to overcome barriers were mapped onto the second tier of the BCW: intervention functions. Themes corresponded with education, persuasion, incentivisation, training,environmental restructuring.

### Education

#### Staff education

Participants emphasised the importance of staff education to increase testing awareness and motivation. Some suggested comprehensive education on testing processes, including how to test, deliver results, and treat infections:

*‘Education, so for the healthcare provider understanding, again, which areas we’re specifically looking at and just making sure we’re all up to date with the latest information and how it’s tested, who’s paying for the testing, how they get their results, what do you do if you need to follow them up?’* (PN, female, aged 51–60 years, interview 18)

A separate participant added that education should take place within the practice and *‘not take very long’*.

#### Leaflets

Staff commonly recommended using leaflets as patient educational tools. They advocated for leaflets with written and visual information on testing to streamline the conversation within time constraints: *‘Generally, people want information to look at ... also if you’re in a very tight consultation there is information for them to read if you haven’t got time to go through it in detail”* (PN, female, aged 51–60 years, interview 18). They also suggested this could increase provider confidence in discussing chlamydia.

### Persuasion

#### Normalising testing offers to patients

Participants recommended normalising chlamydia testing as a standard check to avoid patients feeling singled out: *‘And just to say, this is something we offer to everybody, just like we check everyone’s blood pressure ...’* (GP, female, aged 21– 30 years, interview 3). Normalisation was also cited as a method of reducing shame for patients.

#### Posters

Posters were recommended as a mechanism for stimulating action. One participant stated that consultation room posters could remind staff to offer testing. However, another expressed that the poster had led to desensitisation:

*‘I mean I’ve got the chlamydia poster up in my room but you know when you just see stuff all the time and it doesn’t jump out so it’s not an aide memoire* ...’ (PN, female, aged 51–60 years, interview 19)

Most participants believed posters can prepare patients to expect testing offers, making them *‘subliminally primed for it’* and not caught off-guard.

### Incentivisation

#### Incentives

Staff recommended financial incentives directly to providers, increasing motivation to test. A few participants recommended incentives for patients: one participant stated that a previous initiative providing film tickets to patients helped normalise testing offers:

*‘It was a positive thing to do even though it’s only going to see a film but at least it just made it an everyday word rather than a sexual disease that nobody talks about.’* (PN, female, aged 51–60 years, interview 18)

### Environmental restructuring

#### Computer reminders

Many participants suggested age-related computer reminders to offer testing for patients aged <25 years:

*‘... I might have overlooked an age and so didn’t ask them, so it is good to have that reminder, otherwise it just doesn’t cross your mind.’* (GP, male, aged 41–50 years, interview 4)

While these were often viewed as motivating, some participants admitted to ignoring them owing to desensitisation and ‘*trigger fatigue’* if there are other notifications.

### Policy categories

Some proposed facilitators qualified as policy categories, the third tier of the BCW, which serves to enable intervention functions. Identified policies include communication and marketing and service provision.

### Communication and marketing

#### Advertising tests

Many recommended advertising chlamydia tests through social media. Websites and apps, including Facebook and WhatsApp, were identified as effective in reaching young patients to advertise and create awareness:

*‘Advertising for the younger population, they’re more with WhatsApp and texting, social media, so advertising on there would probably be a way to make them aware or get them worried enough to come in.’* (GP, male, aged 61–70 years, focus group 5)

Social media adverts were also cited to prime patients into expecting testing offers.

#### Campaigns

Participants recommended *‘blanket campaigns’* to normalise testing offers to patients.Social media advertising campaigns were cited as helpful in priming patients to expect testing offers in unrelated consultations:

*‘It’s something that everybody’s doing together, so it’s perhaps something they’re not completely surprised by bringing it up in a random consultation because it’s something out there that they know, and that would make it easier.’* (PN, female, aged 51– 60 years, interview 18)

### Service provision

#### Self-tests

Many participants supported self-testing kits. One described it as a method of streamlining the process and placing responsibility on patients:

*‘That’s easy because you can just shove a swab in their hand and tell them to drop it back at reception ... That’s really quick and you don’t necessarily have to do it.’* (PN, female, aged 61–70 years, interview 7)

Participants encouraged discreet access of self-kits, including placements in *‘conspicuous places’* such as toilets or corridors. Staff also recommended self-kits at reception, either in display boxes or offered by receptionists. However, some disagreed and cited privacy concerns, noting it would be *‘really off putting for a lot of young people’*.

Additionally, a few participants stated that receptionists felt uncomfortable offering tests. Staff also noted the convenience of directing patients to online testing, allowing them to send tests and receive results by mail.

#### Drop-in sessions at other venues

Some participants recommended other venues for health promotion, including drop-in sessions at youth centres, schools, and universities.

*‘*[Where] *there are youth centres, if there can be drop-in places there ... If you’ve got young people who are still in education, whether that’s at school, whether that’s at a college, whether that’s at university, I think to have drop-in sessions available in those sorts of situations.’* (NP, female, aged 61– 70 years, interview 14)

These environments provide a method of reaching young people where they spend the most time.

#### Testing in certain appointments

Participants recommended automatically testing at new patient registration: *‘I think it should be done when patients register, because then it’s always, like, as part of registering you need to do a chlamydia screening, in that age group’* (GP, female, aged 31– 40 years, interview 15). However, staff mostly recommended chlamydia testing be compulsory in sexual health-related appointments such as contraceptive and cervical smear appointments.

#### Nurses testing patients

One participant recommended a nurse-led service owing to workforce pressures: *‘There’s not enough appointments, not enough doctors, not enough time allocated ... I’m wondering if a nurse-led service sounds quite good’* (GP, female, aged 31–40  years, interview 6). A nurse added that they are better suited for testing because they can build rapport and trust: *‘The doctors now seem to all do shorter days so the patients say, “The only ones who are always here are yourselves”’* (ANP, female, aged 41–50 years, interview 20).

## Discussion

### Summary

We used a new analytical perspective to explore general practice staff’s perceptions of barriers and potential interventions to opportunistic chlamydia testing, and policies for enhanced implementation. The main barriers identified include lack of awareness and engagement with the NCSP, knowledge of the testing process, time constraints, concern around patient reactions, and considering it out of place.

The effects of reduced funding and loss of momentum of NCSP contributed to apathy and disengagement with chlamydia testing. A lack of awareness and promotion may have led to lower prioritisation as staff perceive testing as less critical and unsupported. Participants expressed the lack of NCSP communication needs to be addressed to encourage engagement and maintain motivation for testing. Strategies could include the following: clear communication (for example, about the programme’s goals, guidelines, importance, role of primary care); training (for example, integrating into routine care, stigma reduction, identifying need); resources and implementation support (for example, kits, patient information); and feedback (for example, testing rates, patient outcomes, programme impact).

Barriers include funding cuts and prioritisation of long-term conditions such as diabetes, financially incentivised through the Quality Outcomes Framework.^49^ Between 2013–2014 and 2022–2023, local authority spending on sexual health fell 16.9%.^50^ Increased funding would ensure practices have the resources required; however, given current financial pressures, and already overstretched sexual health services, addressing barriers in general practice remains crucial.^51^

Participants highlighted the lack of access for marginalised people such as rural and LGBTQIA+ communities. This is unsurprising: ranked Europe’s best for LGBTQIA+ rights in 2015, the UK dropped to sixteenth by 2024.^52^ One study found 17% of LGBTQ+ people reported discrimination at general practice,^53^ while 45% of trans people and 55% of non-binary people reported their GP lacked understanding of their needs.^54^ A rural clinic professional expressed frustration at limited sexual health clinics and chlamydia testing. Accessible, inclusive health care, which reduces stigma, prioritises prevention, and improves outcomes, is crucial, especially for those unable to attend general practice. A ‘one-sized’ approach is unsuitable.

Staff highlighted difficulty in raising testing during unrelated appointments but supported offering during contraception checks and cervical screening. In England, cervical screening is offered to all women and people with a cervix registered with a GP who are aged 25–64 years. People aged 25–49 years receive invitations every 3 years, while people aged 50–64 years are invited every 5 years.^55^ Offering chlamydia testing during contraception checks and cervical screening would only benefits people with a cervix whereas men have lower testing rates in general practice.^2^ The NCSP covers only those aged <25 years assigned female at birth,^8^ so contraception checks are better for engaging eligible individuals than cervical screening. Focusing solely on people with a cervix reinforces gendered stigma, perpetuates stereotypes that women are responsible for STI prevention and management, and minimises men’s role. It exacerbates disparities as men are less likely to seek care and potentially leaves men undiagnosed and untreated, continuing transmission. A gender-neutral approach promotes equity, normalisation, and reinforces shared responsibility.

Identified interventions include staff education, poster incentives, and reminders. While traditional media remains useful in the digital age, allowing for wide coverage,^56^ participants noted constant exposure may desensitise staff. Normalisation and universal offers were seen as key to reducing stigma, which adolescents prefer^57^ and aligns with NCSP goals,^4^ suggesting the need for clear communication and understanding of NCSP guidance. Policy recommendations include drop-in sessions at youth centres, schools, and universities. Self-tests at practice receptions were proposed, although some raised privacy concerns and receptionist discomfort. Many proposed interventions and policies targeted patient behaviour change: posters, leaflets, and campaigns were recommended to increase patient awareness. However, research shows patients expect staff to offer tests.^16,17^ Staff discomfort can be addressed through training, peer support, using clear language, role-playing communication skills exercises, and keeping up to date with guidelines, which will in turn promote normalisation.

### Strengths and limitations

Key strengths are the incorporation of interviews and focus groups with a large number of diverse staff, recognising that clinical and non-clinical staff play roles in care pathways. Integration of data across methods strengthens findings by providing enhanced descriptions.^58,59^ Face-to-face interviews allow social cues to be gauged and tend to be spontaneous, while telephone interviews provide access to varying geographic locations, busy individuals, and those who are uncomfortable discussing sensitive topics face to face.^60^ Focus groups stimulate discussion, and sharing and refining of ideas through interaction, which creates a diverse range of perspectives and deeper, nuanced understandings.^61^ In addition, using behavioural theory supports the design of comprehensive interventions to target specific behaviours.

The findings are limited in that the sample is not representative (and representativeness is not the goal of qualitative research). Several participants reported previous experience (and hence a presumed interest) in sexual health. Many responders were White British and female, and their experiences may not be universal. The primarily urban settings of our participants’ practices may affect access to resources and their patient populations, potentially limiting the understanding of the challenges encountered by those in rural or underserved areas.

This research was conducted before the COVID-19 pandemic and NCSP changes to only screen people assigned female at birth. The experiences included here may not fully reflect the current system, but much can still be learnt from them. This is especially the case for new barriers that emerged owing to COVID-19, with a fall in GP consultations during the first lockdown and increased use of remote consultations.^62^

### Comparison with existing literature

Consistent with the literature, this research identifies barriers of low perceived knowledge^13,14,18,22^ and staff discomfort with testing in unrelated appointments.^12,45–48^ Staff cited patient reactions^12^ and lack of feedback^47^ as barriers. Additional barriers echoed here include time constraints,^11,12,45–50^ lack of support for partner notification,^12,45,46,48,49^ and difficulties offering men tests.^19,20^ This may stem from fewer clinical opportunities (men consult less with GPs in England compared with women),^63^ men’s lower engagement with preventive health (linked to traditional masculinity discouraging help-seeking),^64^ and staff assumptions about patient risk, comfort, and priorities.^65^

The reiteration of these barriers shows we have not yet overcome them, emphasising the importance for psychological theory in designing behaviour change interventions. New barriers include effects of reduced funding, perceived NCSP momentum loss, and impacts on marginalised individuals. Identified intervention functions consistent with previous literature include staff education,^14,18,23^ leaflets,^18,19,65^ and reminders.^19,23^ Policy facilitators, such as self-sampling kits^14,23,66^ testing in sexual health appointments,^18^ and nurse-led services,^12^ were also supported. For normalisation,^13,67^ staff recommended advertising through social media,^13^ national campaigns,^13,18,66^ and drop-in clinics at schools^16^ and, going beyond previous suggestions, other locations where young people spend their time.

### Implications for research and practice

These findings highlight the need for multi-component behaviour change interventions to increase general practice chlamydia testing. Between 2022 and 2023 there was a 10.9% decrease in internet-based testing and, for women aged 15–24 years, a 5.6% increase in face-to-face testing.^2^ While internet-based testing expands access and convenience for some,^68^ it can widen health inequalities,^69^ particularly if options are limited or inaccessible. It relies on individuals seeking it out and recognising their need for testing; hence, in-person opportunistic services remain essential.

Recommendations include increased training around testing in general practice and greater NCSP involvement. Training should include clinical and non-clinical staff, fostering a whole-practice self-reinforcing approach, with testing high on the agenda within the general practice environment, ensuring that momentum is continued within the practice itself. Participants noted issues with desensitisation to frequent presentation of repeated information, suggesting that novel and personalised interventions, such as individualised feedback on practice performance is needed,^19^ especially to reinforce key information and reduce onus on patients. Feedback is effective in aligning clinical actions and policy, as seen in a study of GPs’ adherence to urinary tract infection guidelines in Germany, where concise and accessible feedback during consultations was preferred.^70^ Providing self-test kits in conspicuous locations might be effective, requiring minimal clinician time to signpost during busy or unrelated consultations.

The reported lack of access for rural and LGBTQIA+ individuals is critical for policy. We need to expand access for these communities, so future interventions must be applicable to marginalised communities. Given the rapid changes to practice in the wake of the COVID-19 pandemic, especially increased use of telehealth^62^ and internet-based testing, future research should consider how online tests can be leveraged alongside other facilitators to increase access.

A multi-faceted approach embedded in behavioural theory is needed. Media campaigns can normalise sexual health discussions while practice-based interventions can promote testing. Increased funding and heightened awareness of NCSP are crucial to sustaining effective interventions. Without sufficient resources, efforts to address barriers risk losing impact and momentum, and chlamydia will remain a significant public health burden.

## Data

Data for this study are not publicly available.
